# Comparison of gene mutation spectrum of thalassemia in different regions of China and Southeast Asia

**DOI:** 10.1002/mgg3.680

**Published:** 2019-04-09

**Authors:** Zhuo Yang, Quexuan Cui, Wenzhe Zhou, Ling Qiu, Bing Han

**Affiliations:** ^1^ Department of Clinical Laboratory Peking Union Medical College, Chinese Academe of Medical Science Beijing China; ^2^ Department of Hematology Peking Union Medical College, Chinese Academe of Medical Science Beijing China

**Keywords:** China, comparison, gene mutation, Southeast Asia, thalassemia

## Abstract

**Background:**

Thalassemia is a common genetic disorder. High prevalence of thalassemia is found in South China, Southeast Asia, India, the Middle East, and the Mediterranean regions. Thalassemia was thought to exist only in southern China, but an increasing number of cases from northern China have been recently reported.

**Methods:**

During 2012 to 2017, suspected thalassemia people were detected for common α‐ and β‐thalassemia mutations by gap‐Polymerase Chain Reaction (PCR) and reverse dot blot (RDB) analysis in Peking Union Medical College Hospital. One thousand and fifty‐nine people with thalassemia mutations were analyzed retrospectively. We picked mutated individuals who originally came from northern areas, and conducted telephone follow‐up survey in order to collect their ancestral information. Besides, we used “thalassemia”, “mutation”, and “Southeast Asian countries” as keywords to search the relevant studies in PubMed and Embase databases.

**Results:**

All carriers included in our study were resided in northern China. Among them, 17.3% were native northerners and 82.7% were immigrants from southern China. Although substantial difference was found in α‐ and β‐thalassemia ratio and detailed spectrum of α‐ and β‐globin mutation spectrum between our data and data obtained from a previous meta‐analysis literature focused on southern China, the most common gene mutations were the same. Similar β‐thalassemia mutation spectrum was found among Thai, Malaysian Chinese, and Guangdong people, however, no other similarities in gene profile were found between Chinese and other ethnic groups in Southeast Asia.

**Conclusion:**

Chinese people in different areas had similar gene mutation, whereas they had significantly different mutation spectrums from other ethnic groups in Southeast Asia.

## 1 INTRODUCTION

Thalassemia is a common monogenic disease including two major types, α‐ and β‐thalassemia, according to mutations or deletions in α‐ and β‐globin genes (HBA1, HBA2 and HBB; OMIM: 141800, 141850 and 141900), respectively. The mutated or deleted genes produce impaired globin protein subunits and affect oxygen transportation. As estimated by a previous research, at least 20% of the world population carry α^+^‐thalassemia, and 5.2% of the population carry a significant variant of β‐thalassemia and α^0^‐thalassemia, who typically have microcytosis and may have mild anemia as well (Modell & Darlison, 2008). However, the distribution of thalassemia is not homogeneous. High prevalence of thalassemia was mainly reported in southern China, Southeast Asia, India, the Middle East, Africa and Mediterranean region (Li, 2017). As reported by Kuesap, Chaijaroenkul, Rungsihirunrat, Pongjantharasatien, and Na‐Bangchang (2015), thalassemia has protective effects on the development of hyperparasitemia and severe anemia in malaria patients, which may justify why the prevalence of thalassemia is high in malarious areas.

Thalassemia is also common in China. Nearly all patients with thalassemia come from the southern China, however, an increasing number of sporadic cases who belong to northern China have been reported (Gan, Yang, Chen, Zhang, & Cui, 2014). We have found a few cases of thalassemia patients whose ancestral home was north China as well. Although immigration from south to north always happens, however, de novo mutations can also happen in people who originated in the northern area. The actual rate of prevalence of thalassemia in northern China is not currently clear, however, we believe that adequate knowledge about thalassemia symptoms and gene distribution can help clinicians screen and diagnose thalassemia candidates (Lai, Huang, Li, & He, 2017). Apart from geographical factors, ethnic difference may be another important factor for the gene diversity.

In this study, we compared mutation spectrum of α‐ and β‐thalassemia in indigenous northern people and immigrants from southern China, and also compared the mutation spectrum of immigrants from southern China with the largest meta‐analysis in southern China, and the data belong to Southeast Asian countries. The results may help understand the similarities and differences among people who came from different areas and ethnic groups.

## 2 METHODS

### 2.1 Genetic testing

Venous blood samples from 2,136 cases who were suspected of thalassemia were collected and tested in Peking Union Medical College Hospital (Beijing, China), the largest place for thalassemia gene analysis in northern China, from 2012 to 2017. Blood tests included blood cell analysis, biochemical parameters, hemoglobin electrophoresis, and thalassemia mutation detection using gap‐PCR and reverse dot blot (RDB) analysis.

In order to test the mutation or deletion of thalassemia, we collected 5 ml venous blood from each patient. We used QIAamp DSP DNA Blood Mini Kit to extract DNA from blood. Thirty‐five cycles of PCR were performed with index primers. Three common HBA1 deletions were tested by gap‐PCR compared to positive controls by electrophoresis through 1.2% agarose gels. Three common HBA2 mutations and 17 mutations of HBB were tested through RDB analysis. The amplification products were denatured, spotted onto nylon membranes and then hybridized with specific oligonucleotide probes. All the deletions or mutations we tested are shown in Table 1.

**Table 1:** Target mutations in this study

	Gene	Mutation	HGVS name
α‐globin deletions	−α^3.7^	−α^3.7^	NG_000006.1:g.34164_37967del3804
	−α^4.2^	−α^4.2^	HGVS not attributable
	‐‐^SEA^	‐‐(SEA)	NG_000006.1:g.26264_45564del19301
α‐globin mutations	α^CS^	Hb Constant Spring (Hb CS)	HBA2:c.427T>C
	α^QS^	Hb Quong Sze	HBA2:c.377T>C
	α^WS^	Hb Westmead	HBA2:c.369C>G
β‐globin mutations	CD41/42	Codons 41/42 (‐TTCT)	HBB:c. 124_127delTTCT
	CD43	Codon 43 (G‐>T)	HBB:c.130G>T
	IVS‐II‐654	IVS‐II‐654 (C‐>T)	HBB:c.316‐197C>T
	−28	−28 (A‐>C)	HBB:c.‐78A>C
	−29	−29 (A‐>G)	HBB:c.‐79A>G
	−30	−30 (T‐>C)	HBB:c.‐80T>C
	−32	−32 (C‐>A)	HBB:c.‐82C A
	CD71/72	Codons 71/72 (+A)	HBB:c.216_217insA
	βE	Hb E	HBB:c.79G>A
	17	Codon 17 (A‐>T)	HBB:c.52A>T
	CD14/15	Codons 14/15 (+G)	HBB:c.45_46insG^a^
	CD31	Codon 31 (‐C)	HBB:c.94delC
	CD27/28	Codons 27/28 (+C)	HBB:c.84_85insC
	IVS‐I‐1	IVS‐I‐1 (G‐>A) IVS‐I‐1 (G‐>T)	HBB:c.92+1G>A HBB:c.92+1G>T
	IVS‐I‐5	IVS‐I‐5 (G‐>C)	HBB:c.92+5G>C
	Cap+1	1. CAP+1 (A‐>C)	HBB:c.‐50A>C
		2. 5'UTR; +43 to +40 (‐AAAC) beta+	HBB:c.‐11_‐8delAAAC
	Initiation Condon	Initiation codon ATG‐>AGG	HBB:c.2T>G

#### 2.1.1 Ancestral home

Using hospital's administration data, 523 individuals who came from northern China, were selected from 1,372 α‐ and β‐thalassemia carriers. Telephone follow‐up survey was conducted on the basis of their ancestral information, including a household registration and prevalence of their ancestors in three generations of family, to confirm whether these northerners have southern lineage. Carriers with no follow‐up results or searchable identity cards were excluded, and 1,059 cases with ancestral information were involved in the final analysis of results.

### 2.2 Literature review

We used “thalassemia”, “mutation”, and “Southeast Asian countries” as keywords to find out relevant studies in PubMed and EMbase updated on 27 December 2017. We considered Southeast Asia which consisted of these countries: Philippines, Malaysia, East Timor, Indonesia, Brunei, Singapore, Cambodia, Laos, Myanmar, Thailand, and Vietnam. The included studies should present in English and cover the majority of the nation. Furthermore, studies on special populations (e.g. minor ethnic groups) were excluded.

### 2.3 Statistical analysis

Statistical analysis was conducted using SPSS 22.0 software (IBM, NY). The ratio of α‐ and β‐thalassemia alleles was calculated. In addition, data from included studies and our study were analyzed by Fisher's exact test and Pearson's chi‐squared test. *p* ＜ 0.05 was statistically considered significant.

### 2.4 Ethical statement

The Ethics Committee of Peking Union Medical College Hospital approved the study (Reference No. S‐K438). All participants provided written informed consent as well.

## 3 RESULTS

### 3.1 Comparison of our thalassemia mutation spectrum in northern and southern China

Data of 1,059 individuals who had thalassemia gene abnormalities were collected during November 2012 to July 2017. Among them, 33.9% (359/1,059) were α‐thalassemia carriers or patients, with 330 carrying one deletion or mutation, and 29 carrying two deletions or mutations; 64.5% (683/1,059) were β‐thalassemia carriers or patients, with 682 carrying one mutation, and 1 carrying two mutations; 1.6% (17/1,059) were both α‐ and β‐thalassemia carriers or patients. All these people had settled in northern China for more than 3 years.

The ratio of the most common deletions and mutations of α‐thalassemia: ‐‐^SEA^, ‐α^3.7^, ‐α^4.2^, α^CS^, α^WS^ and α^QS^; was 74.8%, 16.1%, 16.1%, 1.7%, 1.2%, and 0.7%, respectively, and the most common five gene mutations of β‐thalassemia were IVS‐II‐654 (36.8%), CD41‐42 (28.3%), CD17 (25.2%), −28 (3.0%), and CD71‐72 (2.8%), respectively.

People were classified as indigenous northern population (17.3%) and southern immigrants (82.7%), depending on their ancestral home (Table 2). Since there was no significant difference in individual gene distribution between the two groups either in α‐thalassemia (*p* = 0.221) or β‐thalassemia (*p* = 0.979), we considered people from northern China and southern China together as a whole in comparison with people of Chinese meta‐analysis and Southeast Asia.

**Table 2:** Frequency and distribution of α‐ and β‐thalassemia mutations

Mutation	Northern population		Immigrants from southern region		Total	
	*N*	%	*N*	%	*N*	%
α‐thalassemia						
Total	49	100.0	356	100.0	405	100.0
‐‐^SEA^	35	71.4	268	75.2	303	74.8
‐α^3.7^	12	24.5	53	14.9	65	16.1
‐α^4.2^	2	4.1	20	5.6	22	5.4
α^CS^	0	0	7	2.0	7	1.7
α^QS^	0	0	5	1.4	5	1.2
α^WS^	0	0	3	0.9	3	0.7
χ^2^ test	χ^2^=2.582, *p* = 0.275					
β‐thalassemia						
Total	131	100.0	541	100.0	672	100.0
IVS‐II‐654 (C‐T)	52	39.7	195	36.0	247	36.8
CD41/42 (‐TTCT)	33	25.2	157	29.0	190	28.3
CD17 (A‐T)	35	26.7	134	24.8	169	25.3
−28 (A‐G)	2	1.5	18	3.3	20	3.0
CD71/72 (+A)	3	2.3	16	3.0	19	2.8
CD27/28 (+C)	1	0.8	6	1.1	7	1.0
CD43 (G‐T)	1	0.8	6	1.1	7	1.0
βE (GAG‐AAG)	2	1.5	4	0.7	6	0.9
−29 (A‐G)	1	0.8	3	0.6	4	0.6
CD14/15 (+G)	0	0	1	0.2	1	0.1
IVS‐I‐1 (G‐T/G‐A)	0	0	1	0.2	1	0.1
IVS‐I‐5 (G‐C)	1	0.8	0	0	1	0.1
χ^2^ test	χ^2^ = 2.409, *p* = 0.661					

### 3.2 Comparison of our thalassemia mutation spectrum data and a former meta‐analysis in the Chinese population

To date, the largest meta‐analysis of thalassemia mutation spectrum in China has been conducted by Lai et al. (Sayani & Kwiatkowski, 2015). Most of the data are obtained from people who settled in the southern China. Our data is the first one about people in northern China.

For α‐thalassemia, although the detailed gene compositions showed significant difference (*p* ＜ 0.001) between our data and Lai et al.’s data, the first three most common mutations, which accounted for nearly 90% of patients, were the same: ‐‐^SEA^ ranked first, ‐α^3.7^ ranked second and –α^4.2^ ranked third in both groups, respectively (Table 3). The results of patients originated from southern China and the previous meta‐analysis were significantly different (*p* ＜ 0.001). However, while comparing indigenous northern population and the previous meta‐analysis, we found that they were not significantly different (*p* = 0.065).

**Table 3:** The thalassemia gene distribution in our study and the literature among Chinese people

	Our data	Meta‐analysis conducted by Lai et al. (Sayani & Kwiatkowski, 2015)
α‐thalassemia		
*N*	405	5,806
‐‐SEA	303 (74.8%)	2,958 (51.0%)
‐α^3.7^	65 (16.1%)	1598 (27.5%)
‐α^4.2^	22 (5.4%)	554 (9.5%)
α^CS^	7 (1.7%)	249 (4.3%)
α^QS^	5 (1.2%)	92 (1.6%)
α^WS^	3 (0.7%)	355 (6.1%)
*p* value	<0.001	
β‐thalassemia		
*n*	701	2,403
IVS‐II−654	258 (36.8%)	448 (18.6%)
CD41/42	204 (29.1%)	931 (38.7%)
CD17	176 (25.1%)	387 (16.1%)
CD71/72	19 (2.7%)	83 (3.5%)
−28	17 (2.4%)	262 (10.9%)
βE	4 (0.6%)	68 (2.8%)
Other	23 (3.3%)	224 (9.3%)
*p* value	<0.001	

For β‐thalassemia, IVS‐II‐654 was the most frequent allele in our study, while CD41/42 gene ranked first in Lai et al.’s study (Table 3). Nevertheless, similar to α‐thalassemia, the first three common mutations were IVS‐II‐654, CD41/42, and CD17 in both studies, which accounted for more than 70% of the cases (the five most frequent alleles of β‐thalassemia were listed, and other less frequent alleles were classified as “other”). Analysis of gene constitutions showed significant difference between the overall population of our study and the Lai et al.’s study (*p* ＜ 0.001). Besides, we compared meta‐analysis result with our northern (*p* ＜ 0.001) and southern (*p* ＜ 0.001) subgroups, and the result observed showed that either of them had significant difference.

### 3.3 Comparison of our data with data obtained from different Chinese provinces in the literature

We found out all the origin papers from Lai et al.’s meta‐analysis (Cai et al., 2002; Chen, Chen, Xia, & Qin, 2004; Li, Lan, & Luo, 2009; Pan, Long, & Li, 2007; Qiu, Chen, & Zhang, 2009; Xiong et al., 2010; Xu et al., 2013; Yao, Yu, et al., 2013; Yao, Zhang, et al., 2013; Yin et al., 2014; Zeng, Chen, Chen, Zhong, & Qiu, 2014; Zhang, Wang, & Gao, 2010). Among them, nine studies involved α‐thalassemia and seven involved β‐thalassemia gene distribution. We compared allele component between immigrants from southern China in our group and four provinces in southern China that were included in Lai et al.’s meta‐analysis (Table 4). For α‐thalassemia, our data were similar to that of Fujian province, while that were different from provinces of Guangdong, Guangxi, and Chongqing, which reflected relatively higher ratio of ‐α^3.7^. For β‐thalassemia, mutation spectrum was consistent in our group and the four provinces in southern China as mentioned in the literature (Table 4).

**Table 4:** α‐ and β‐thalassemia gene distribution in four southern provinces

	Fujian province				Guangdong province				Guangxi province				Chongqing province			
	Our data		Literature (Xu et al., 2013)		Our data		Literature (Chen et al., 2004; Yin et al., 2014; Zhang et al., 2010)		Our data		Literature (Cai et al., 2002; Li et al., 2009; Pan et al., 2007; Qiu et al., 2009; Xiong et al., 2010; Zeng et al., 2014)		Our data		Literature (Yao, Yu, et al., 2013; Yao, Zhang, et al., 2013)	
	*n*	%	*n*	%	*n*	%	*n*	%	*n*	%	*n*	%	*n*	%	*n*	%
α‐thalassemia																
Total (*N*)	40		363		33		3,889		83		1722		4		56	
‐SEA	28	70.0	240	66.1	28	84.9	2001	51.5	58	69.9	784	45.5	3	75.0	10	17.9
‐α^3.7^	9	22.5	75	20.7	3	9.1	1,104	28.4	13	15.7	437	25.4	0	0.0	30	53.6
‐α^4.2^	0	0.0	26	7.2	1	3.0	371	9.5	8	9.6	194	11.3	1	25.0	5	8.9
α^CS^	0	0.0	5	1.4	1	3.0	102	2.6	3	3.6	136	7.9	0	0.0	0	0.0
α^QS^	2	5.0	12	3.3	0	0.0	52	1.3	1	1.2	96	5.6	0	0.0	1	1.8
α^WS^	1	2.5	2	0.6	0	0.0	252	6.5	0	0.0	52	3.0	0	0.0	0	0.0
χ^2^	5.98				14.079				18.803				8.267			
*p*	0.241				0.009				0.001				0.026			
β‐thalassemia																
Total (*N*)	29		148		35		1,201		66		1,009		15		45	
IVS‐II‐654	15	51.7	65	43.9	9	25.7	312	26.0	4	6.1	67	6.6	6	40.0	9	20.0
CD41/42	10	34.5	40	27.0	15	42.9	471	39.2	28	42.4	427	42.3	7	46.7	21	46.7
CD17	3	10.3	12	8.1	6	17.1	98	8.2	24	36.4	283	28.1	2	13.3	5	11.1
CD71/72	0	0.0	10	6.8	3	8.6	170	14.2	6	9.1	78	7.7	0	0.0	0	0.0
−28	0	0.0	1	0.7	0	0.0	14	1.2	4	6.1	65	6.4	0	0.0	0	0.0
βE	0	0.0	2	1.4	0	0.0	31	2.6	0	0.0	36	3.6	0	0.0	5	11.1
−29	0	0.0	2	1.4	2	5.7	19	1.6	0	0.0	7	0.7	0	0.0	4	8.9
Other^a^	1	3.5	16	10.8	0	0.0	86	7.2	0	0.0	46	4.6	0	0.0	1	2.2
χ^2^	4.673				9.525				6.896				4.409			
*p*	0.693				0.151				0.391				0.505			

a All other rare mutations in β‐thalassemia were categorized as “other.”

### 3.4 Comparison of thalassemia mutation spectrum in China and different Southeast Asian countries

**Figure 1:**
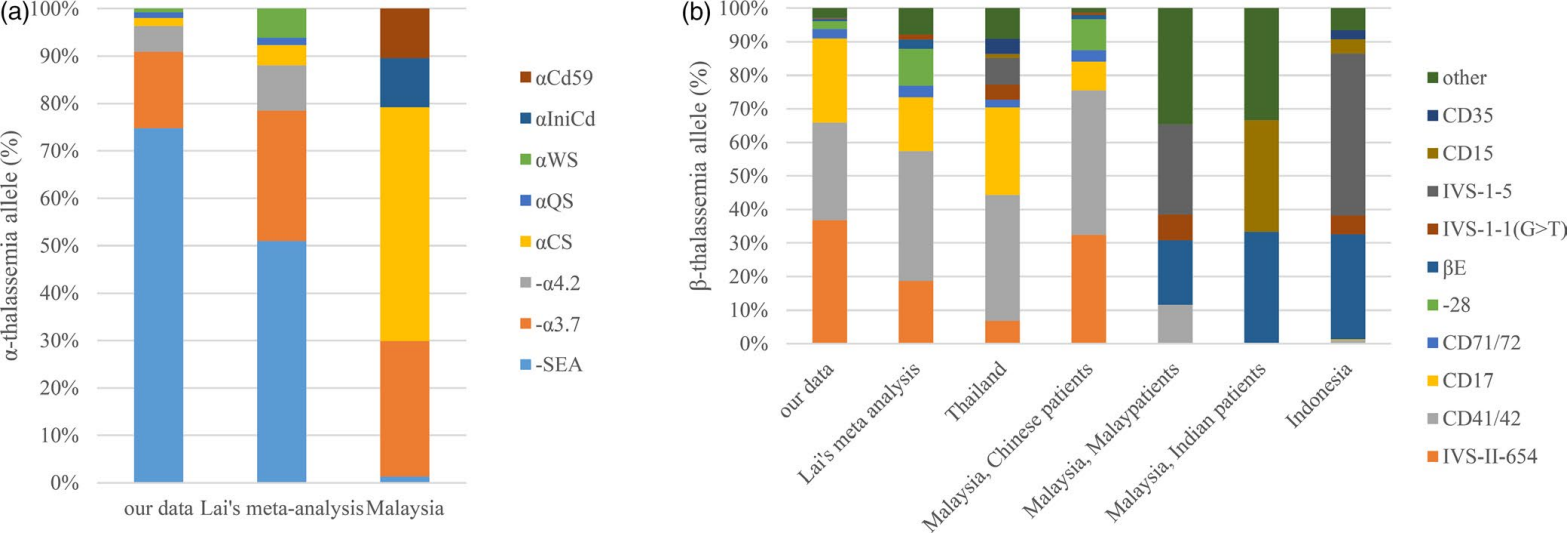
Comparison of mutation spectrum between China and Southeast Asia. (a) α‐thalassemia and (b) β‐thalassemia

We next compared our data of immigrants from Southern China, collected data from previous Chinese reports, and the data collected from literature focused on Southeast Asia for gene distribution. Studies in Thailand (Boonyawat, Monsereenusorn, & Traivaree, 2014), Indonesia (Rujito, Basalamah, & Mulatsih, 2015), and Malaysia (Dxr et al., 2017; Tan et al., 2006) were finally selected (Figure 1) according to our criteria. The Malaysian report offered gene composition for α‐thalassemia and different ethnic data, which included Chinese, Malay, and Indian people for β‐thalassemia.

For α‐thalassemia, there were few data from Southeast Asia. Different from Chinese people (including our data), in whom ‐‐^SEA^, ‐α^3.7^, ‐α^4.2^ were the most common alleles. Malaysians had a high ratio of α^CS^ (44.19%) and sizable proportions of α^IniCd^ (9.30%) and α^Cd59^ (9.30%), which were rarely seen in Chinese people (Figure 1a).

For β‐thalassemia, Thai and Malaysian Chinese people had similar frequent genes (IVS‐II‐654, CD41/42, and CD17) with Chinese people. Detailed analysis showed that Malaysian Chinese individuals had the same mutation spectrum as Guangdong people, both in our study (*p* = 0.135) and Lai et al.’s research (*p* = 0.068), indicating a possible similar race origin. However, for Indonesian, Malay, and Indian people in Malaysia, the gene constitution was very different from that of Chinese. They had a high percentage of βIVS‐5 mutation, which was almost not found in Chinese (Figure 1b).

## 4 DISCUSSION

As far as we know, this is the first study that compared α‐ and β‐thalassemia mutation spectrum of people who came from northern and southern China and Southeast Asian countries.

As shown in our study, people came to our hospital to detect thalassemia gene mainly due to microcytic hypochromic anemia, hemolytic anemia of unknown origin, or positive family history of thalassemia. Although there were no strict demographic census data, our study reflected the thalassemia gene distribution in north China to some extent because our hospital is the largest center for thalassemia gene screening in north China.

There was more β‐thalassemia patients than α‐thalassemia patients in our study, which is consistent with some of the thalassemia epidemic literature in China (Niu, Huang, An, Wang, & Jiang, 2016; Zhang et al., 2012). However, Lai et al.’s data were in agreement with studies conducted in Taiwan (Peng et al., 2013) and Guilin, Guangxi (Tang et al., 2015). β‐thalassemia was distributed in wide regions, such as Southeast Asia, the Middle East, the Mediterranean region, and North and South America. However, cases of α‐thalassemia were mostly observed in Southeast Asia, the Middle East, Africa and Mediterranean region (Kaushansky, Beutler, & Kipps, 2010). We presumed that the distribution of α‐ and β‐thalassemia would be different in China as well. Lai et al.’s meta‐analysis included four provinces (as shown in Table 4), while our study covered nearly all provinces of mainland China. We had a significant part of patients who originated from provinces of Sichuan, Hunan, and Hubei, which was not included in Lai et al.’s study. In our study, the percentage of β‐thalassemia was 72.3%, 71.2% and 73.2% in Sichuan, Hunan, and Hubei, respectively, which indicated that there were more β‐thalassemia patients in these provinces. The difference of relative number of α‐ and β‐thalassemia between ours and Lai et al.’s data was probably due to the difference of samples. We hypothesized that α‐thalassemia patients mainly concentrated in Southern coastal region of China.

Difference of α‐thalassemia mutation spectrum between northern China (our data) and southern China (Lai et al.’s data) was statistically significant (*p* < 0.001). We noticed that patients who lived in the north had higher percentage of ‐‐^SEA^ than those who originated from the same province, but lived in southern China. This phenomenon was observed in the provinces of Fujian (Xu et al., 2013), Guangdong (Chen et al., 2004; Yin et al., 2014; Zhang et al., 2010), Guangxi (Cai et al., 2002; Li et al., 2009; Pan et al., 2007; Xiong et al., 2010; Zeng et al., 2014), and Chongqing (Yao, Yu, et al., 2013) (Table 4). The high gene frequency of ‐‐^SEA^ in northern China could be partly explained by the difference of sampling methods. In Lai et al.’s meta‐analysis, all included studies used cluster sampling or stratified sampling, while in our study, only suspected thalassemia patients were involved in genetic testing. It is known that people with only one ‐α^3.7^ or –α^4.2^ deletions were silent carriers, and may not have clinical manifestations or anemia. Thus, probably less carriers with ‐α^3.7^ or –α^4.2^ were included in our study, and the percentage may decrease as well. Furthermore, our study covered nearly all the provinces in mainland China, and the proportion of people from some provinces was different from Lai et al.’s meta‐analysis. Even though, the three most common mutations, which accounted for nearly 90% of patients, were the same in ours and Lai et al.’s report, indicating the consistency of gene pattern of Chinese people.

Similarly, the three most common mutations of β‐thalassemia in our group, IVS‐II‐654, CD41/42 and CD17, were similar to those of Lai et al.’s data although the ratios of them were different. Nevertheless, we did not observe the difference in the four provinces that we chose to analyze in both groups. β‐Thalassemia mutation spectrum was also different in different provinces. For example, IVS‐II‐654 was the most common mutation in Fujian, while CD41/42 was common in the other three provinces. Consequently, the difference between our data and Lai et al.’s data was probably due to difference in samples collected from different provinces. Many provinces were not included in the detailed comparison because data of these provinces were absent in Lai et al.’s study.

The mutation spectrum of α‐ and β‐thalassemia is diverse in different areas, however, the race difference may also play a great role. Although Chinese and Southeast Asian people were both yellow races, they had significant difference in thalassemia mutation spectrum. For α‐thalassemia, the most common allele in Malaysia was α^CS^ (44.19%) instead of ‐‐^SEA^ in China. We also paid attention to the sizable proportions of α^IniCd^ (9.30%) and α^Cd59^ (9.30%) in Malaysians, which were rare in Chinese patients. As for β‐thalassemia, IVS‐II‐654, a common mutation in China, was not very common in Southeast Asia countries, except for Chinese Malaysians. IVS‐I‐5 and βE were common in Malaysia and Indonesia (Rujito et al., 2015; Tan et al., 2006), but not in China.

Interbreeding of indigenous people and immigrants may increase the diversity of local mutation spectrum. For instance, HbS mutation in Central West Africa flew to North Africa due to the Ottoman rule in the 17th century, and then flew to Europe through slavery roads. As a result, thalassemia patients in the Mediterranean region have a high ratio of Codon 39 (C→T) and IVS‐I‐110 (G→A) mutation in HBB, which are the most frequent gene in Tunisia and Algeria (Anwar, Khyatti, & Hemminki, 2014). Migration from China to Southeast Asia altered the mutation spectrum of thalassemia. As shown in Figure 1b, Chinese people and Malaysian Chinese had similar gene distribution diagram. Detailed analysis even showed that for β‐thalassemia, Malaysian Chinese had the same mutation spectrum as Guangdong people, indicating a possibly similar race origin. Since Ming Dynasty, a large number of Chinese people had immigrated to Southeast Asia, and most of them originated from coastal areas, including Guangdong province. Similar explanation may also be true for Thai people, although the study that we chose did not clarify the ration of patients, thus we could only see the overall gene distribution instead of the separated ones (Boonyawat et al., 2014).

In addition to Chinese people, Indians were also a large immigrant group in Southeast Asia (Wu, 2015), which also made an impact on thalassemia mutation spectrum in Southeast Asia. The most frequent allele of α‐thalassemia in Indians was –α^3.7( ^Purohit, Dehury, Patel, & Patel, 2014), which was observed in Malaysians as well. As for β‐thalassemia, IVS‐I‐5 (G → C), Codon 15 (G → A), and Codon 30 (G → C), which were common in India (Edison et al., 2008), were also seen in Thailand, Malaysia, and Indonesia. However, IVS‐I‐5 mutation was also observed in Malay patients, thus it is not clear whether this mutation was originated from native people or Indian immigrants.

It is the first time that we describe the similarities and differences between Chinese people in different areas and Chinese people with other races in Southeast Asia. Further studies with larger sample size and more included countries with human species composition are helpful for the complete understanding of gene transition in these countries and races as well.

## CONFLICT OF INTEREST

The authors declare that there are no conflict of interests regarding the publication of this article.
