# Comparison of gene mutation spectrum of thalassemia in different regions of China and Southeast Asia

**DOI:** 10.1002/mgg3.1018

**Published:** 2019-11-03

In Yang et al., 2019, the affiliations of the authors were published with error. “Peking Union Medical College” should have been "Peking Union Medical College Hospital.”

The correct author listing and their affiliations are, as follows:

**Zhuo Yang^1^** | **Quexuan Cui^2^** | **Wenzhe Zhou^2^** | **Ling Qiu^1^** | **Bing Han^2^**

^1^Department of Clinical Laboratory, Peking Union Medical College Hospital, Peking Union Medical College & Chinese Academy of Medical Sciences, Beijing 100730, China

^2^Department of Hematology, Peking Union Medical College Hospital, Peking Union Medical College & Chinese Academy of Medical Sciences, Beijing 100730, China

**Correspondence**

Bing Han, Department of Hematology, Peking Union Medical College Hospital, Chinese Academy of Medical Sciences, Beijing, China.

Email: hanbing_li@sina.com

We apologize for this error.
